# Dementia and the risk of poor outcomes as a result of the COVID-19 pandemic

**DOI:** 10.12968/bjnn.2022.18.5.226

**Published:** 2022-10-31

**Authors:** James Edward Hill, Katalin Ujhelyi Gomez, Joanna Harrison

**Affiliations:** 1 University of Central Lancashire; 2 University of Liverpool; 3 Synthesis, Economic Evaluation and Decision Science Group, University of Central Lancashire

**Keywords:** dementia, COVID-19, systematic review, severe illness, mortality, infection

## Abstract

People with dementia belong to some of the most vulnerable groups and their vulnerability has been augmented by the Covid-19 pandemic. This article provides a critical appraisal and evaluation of a systematic review that has investigated the relationship between dementia and COVID-19 related outcomes.

## Background

More than 50 million people worldwide live with dementia (Alzheimer’s Disease International 2019). It has been proposed that dementia is one of the greatest challenges to modern health and social care (Barros et al. 2020). Individuals with dementia belong to some of the most vulnerable groups, as their survival is dependent upon the support they receive from formal and informal carers (Bökberg et al. 2018). This vulnerability has been augmented by the COVID-19 pandemic (Brown et al. 2020).

People with dementia and their families have been negatively impacted by the health crisis as a result of severe illness and death from infection (Rajagopalan et al. 2022). Additionally, governments in many countries have failed to protect long-term care facilities with high proportions of patients with dementia (Rothan and Byrareddy 2020). The pandemic also had indirect effects through government measures to contain the spread of the virus, which restricted social support and healthcare provision those with dementia depend on (Brown et al. 2020). This has led to increased social isolation, loneliness, mental health difficulties (Cagnin et al. 2020; Cohen et al. 2020; El Haj et al. 2020), and further cognitive impairments (Boutoleau-Bretonnière et al. 2020).

Special attention and support for patients with dementia are essential for two reasons. First, adherence to preventative measures that help reduce the risk of getting infected including hand hygiene, mask wearing, and physical distancing is more difficult to achieve in this patient group (Sharma 2021). Second, people with dementia are at higher risk of developing respiratory infections (Liao et al. 2015), such as pneumonia, which is the most common cause of death (Brunnström and Englund 2009). Thus, there is emerging evidence to show that there is a link between dementia and adverse COVID-19 outcomes (Atkins et al. 2020). A recent systematic review by Hariyanto and colleagues (Hariyanto et al. 2021) gathered evidence to identify the association between dementia and poor outcomes of COVID-19 infection.

## Aims of commentary

The present commentary aims to critically appraise the methods used in the systematic review conducted by Hariyanto et al (2021) and considers the findings in the context of health and social care.

## Methods

A protocol for this systematic review was not pre-registered. A narrow database search was conducted in PubMed and its European version, the Europe PMC from 2019 to October 2020. Additional forward and backward citation tracking of all included studies was undertaken. The review set out to include randomized controlled trials (RCTs), cohort studies, clinical trials, case-cohort studies, and studies with cross-over design. Only studies which compared patients with and without dementia who contracted COVID-19 and studies that reported risk of COVID-19 infection, severe COVID-19, or mortality were included. Titles, abstracts and full texts screening process was not specified. Data extraction and appropriate quality appraisal was performed independently by two authors using the Newcastle–Ottawa Scale (NOS).

A meta-analysis was conducted using a random-effects model (Mantel-Haenszel formula). The main outcome assessed was risk of poor outcome, comparing people with dementia to people without dementia. ‘*Composite poor outcome*’ refers to risk of infection, sever illness, and mortality. A subgroup analysis was undertaken on the individual risk factors of COVID-19 infection, severe COVID-19 and mortality. Begg’s funnel plot analysis was used to explore risk of publication bias.

## Results

The initial search identified 1835 studies in PubMed and 8425 studies in Europe PMC (total 10260). Due to the similarity of these two databases, a high number of duplicates were found. After screening titles, abstracts and full texts, a total of 24 studies were included in the review. Of these, 21 were retrospective cohort, two studies were prospective cohort, while the remaining one study was a case-control study. No RCTs were included. The review categorised the studies into three sub-groups based on outcomes: risk of infection (four studies), severity of illness (five studies), and mortality (15 studies). However, it was unclear how the authors selected particular outcomes in the cases of studies where there was more than one outcome reported. A ‘good’ quality rating was given to all studies based on the NOS.

Substantial heterogeneity was observed for all comparisons. Meta-analysis showed an association between dementia and ‘*composite poor outcome*’ (RR 2.67 (95% CI 2.06 − 3.47), p < 0.00001, I^2^ = 99%). There was no indication of publication bias on visual inspection of funnel plot for ‘*composite poor outcome*’. Sub-group analyses revealed that dementia was associated with higher risk of COVID-19 infection (RR 2.76 (95% CI 1.43 − 5.33), p = 0.003, I^2^ = 99%), severe illness (RR 2.63 (95% CI 1.41 − 4.90), p = 0.002, I^2^ = 89%), and mortality (RR 2.62 (95% CI 2.04 − 3.36), p < 0.00001, I^2^ = 96%).

## Commentary

Based on the Joanna Briggs Institute Critical Appraisal tool for systematic reviews (Aromataris et al. 2015), six out of 11 criteria were judged to be satisfactory for the present review. The main areas of concern are listed as follows. The review searched for studies using only two, very similar databases. This may have led to the introduction of publication bias, which refers to the possible absence of relevant studies. There was no indication of publication bias for risk of poor outcome. However, the visual assessment of the funnel plot was not possible for the outcomes of COVID-19 infection risk, severe illness and mortality due to too few studies.

The review used a meta-analysis to combine the results of the included studies, an appropriate method given the available data. However, it only included one outcome for each study without providing a rationale as to why that particular outcome was extracted, despite the availability of other relevant outcomes e.g. (Barros et al. 2020). Sub-group analyses were performed using the selected outcomes and the results were combined. This may have introduced reporting bias where some outcomes may have been selectively reported depending on the nature and the direction of the results.

Combining the results of the sub-group analyses into one composite outcome is problematic due to the high level of heterogeneity in the included outcomes. The findings would be clearer if meta-analyses had been conducted separately for each outcome (risk of infection, severity of illness, mortality) as opposed to sub-group analyses. Due to the low methodological quality of this systematic review, it is concluded that the true effect may be markedly different from the effect estimated. It is not possible to draw a definite conclusion as to how high the risk is due to the wide confidence intervals. However, the results of the review show substantial, clinically significant risk for people with dementia to become infected with COVID-19, develop a severe illness, or die.

As a result of the cognitive issues present in people with dementia, such as memory problems, safeguarding procedures (e.g. mask wearing) may be difficult to apply (Covino et al. 2020). Hospitalising and medically assisting patients with dementia can pose challenges due to the increased stress patients experience in new environments, as well as due to behavioural issues (Kales et al. 2019). Additionally, the clinical presentation of COVID-19 in individuals with dementia has been suggested to be atypical, characterised by the onset of hypoactive delirium and deteriorating functional status (Bianchetti et al. 2020). These unusual symptoms make it more difficult for nurses to detect COVID-19 infection in people affected by dementia. The substantial clinically significant vulnerability of people with dementia to COVID-19 highlights the need to minimise their exposure to the virus. However, it is important to consider some of the challenges mentioned above and reflect upon alternative methods of safeguarding procedures. This can be very simple adaptations, such as wearing transparent masks to allow better facial recognition and non-verbal communication (Gil and Arroyo-Anlló 2021). Wherever possible it is important to provide early diagnosis, treatment, and support to those with dementia, and their caregivers, to reduce the risk of serious illness and/or death once infected (Alzheimer’s Disease International 2019).

Future research in this area should address the direct mediating factors that make this population more vulnerable to infection and explore preventative measures. Patient characteristics such as age, comorbid conditions, nutritional status, and daily medication can impact the severity of illness from COVID-19, and therefore should be considered in future research. Finally, there is room for further research in investigating the relationship between dementia and COVID-19 related outcomes.

## CPD reflective questions

1. What are the main strengths and weaknesses of this review?
2. Which of the risk factors for poor outcomes mentioned in this commentary have you come across in your practice?
3. How will knowing these risk factors influence your practice to ameliorate some of these risks?

## Key findings

1. Those with dementia are at higher risk for becoming infected with COVID-19 and developing a severe illness.
2. Those with dementia are at higher risk of mortality from Covid 19 infection
3. Research should further investigate the relationship between dementia and COVID-19 outcomes addressing the mediating factors that place people with dementia at higher risk.
